# RWKV-VIO: An Efficient and Low-Drift Visual–Inertial Odometry Using an End-to-End Deep Network

**DOI:** 10.3390/s25185737

**Published:** 2025-09-15

**Authors:** Jiaxi Yang, Xiaoming Xu, Zeyuan Xu, Zhigang Wu, Weimeng Chu

**Affiliations:** 1 School of Aeronautics and Astronautics, Sun Yat-sen University, Guangzhou 510275, China; yangjx87@mail2.sysu.edu.cn (J.Y.); xuxm29@mail.sysu.edu.cn (X.X.);; 2 Department of Electrical, Computer and Biomedical Engineering, University of Pavia, 27100 Pavia, Italy; 3 Department of Mechanical Engineering, National University of Singapore, Singapore 119077, Singapore

**Keywords:** visual-inertial odometry, data fusion, navigation, positioning, multi-sensor

## Abstract

Visual–Inertial Odometry (VIO) is a foundational technology for autonomous navigation and robotics. However, existing deep learning-based methods face key challenges in temporal modeling and computational efficiency. Conventional approaches, such as Long Short-Term Memory (LSTM) networks and Transformers methods, often struggle to handle dependencies across different temporal scales while causing high computational costs. To address these issues, this work introduces Receptance Weighted Key Value (RWKV)-VIO, a novel framework based on the RWKV architecture. The proposed framework is designed with a lightweight structure and linear computational complexity, which effectively reduces the computational burden in temporal modeling. Furthermore, a newly developed Inertial Measurement Unit (IMU) encoder is included to improve the effectiveness of feature extraction using residual connections and channel alignment, allowing the efficient use of historical inertial data. A parallel encoding strategy uses two independently initialized encoders. Features are extracted from different dimensions by this strategy, strengthening the model’s ability to detect complex patterns. Experimental results for publicly shared datasets show that RWKV-VIO prioritizes computational efficiency and lightweight design. It significantly reduces model size and inference time compared to existing advanced methods while achieving top-ranked positioning accuracy among evaluated approaches.

## 1. Introduction

Accurate localization is essential for robot navigation, autonomous driving [1], aircraft navigation, underwater positioning [2], and augmented reality [3,4,5]. Single sensors often fail to ensure accuracy and robustness in complex environments [6,7]. For example, visual sensors perform poorly in low-texture scenes or under changing lighting conditions. The Inertial Measurement Unit (IMU) offers high-frequency motion estimates but accumulates errors over time [8]. To solve these problems, multisensor fusion technology combines the strengths of different sensors to improve accuracy and reliability [9,10,11,12,13].

Visual–Inertial Odometry (VIO) is a multisensor fusion technique that combines visual data and IMU measurements to deliver high-frequency pose estimation with enhanced robustness under challenging conditions. Compared to Visual Odometry (VO) based solely on vision [14,15,16,17], VIO demonstrates stronger robustness in environments with poor lighting or sparse visual features.

**Figure 1:**
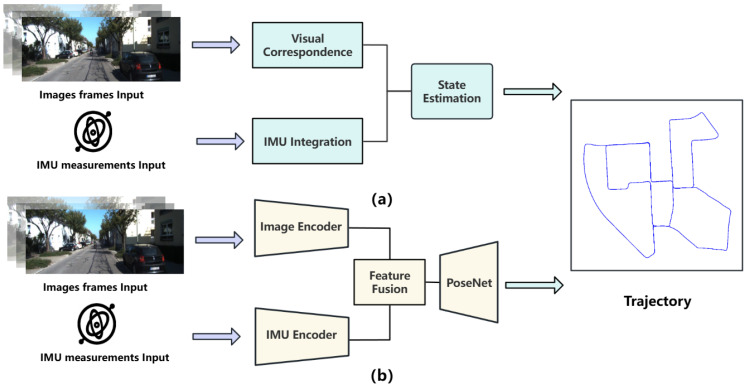
(**a**) VIO methods based on geometric principles. (**b**) VIO methods based on deep learning.

VIO methods can be broadly categorized into two types: geometry-based approaches and learning-based approaches. As shown in Figure 1a, geometry-based methods typically consist of visual correspondence, IMU integration, and state estimation. State estimation, in turn, can be divided into two subcategories: optimization-based and filtering-based methods. Geometry-based approaches [18,19,20] generally achieve high accuracy under controlled or favorable conditions. However, these methods rely heavily on manually designed feature extraction and non-linear optimization techniques, making them highly sensitive to the quality of visual features and the accuracy of initialization. Furthermore, the reliance on manual feature extraction and nonlinear optimization leads to high computational complexity, limiting real-time applicability. In recent years, learning-based VIO models [21,22,23] have shown robustness in complex scenarios by automatically extracting visual and inertial features end-to-end. The general architecture of these methods is shown in Figure 1b, mainly consisting of a visual–inertial encoder, a feature fusion network, and a pose regression network. However, these methods often rely on recurrent neural networks (RNNs), such as Long Short-Term Memory (LSTM) [24] and Gated Recurrent Unit (GRU) [25], for temporal modeling. These models suffer from gradient vanishing problems [26], especially as the sequence length increases. They also struggle to capture long-term dependencies, which reduces accuracy. In addition, their sequential processing prevents parallel computation [27], leading to slower training and inference and limiting scalability.

Deep learning-based VIO models also encounter challenges in extracting meaningful IMU features. Simpler architectures, such as Convolutional Neural Networks (CNNs), often fail to capture long-term dependencies and lose historical information over time. These limitations affect the accuracy of long-duration pose estimation and contribute to accumulated drift in predicted trajectories.

To address these challenges, RWKV-VIO is proposed as a framework based on the RWKV architecture [28]. RWKV is a lightweight temporal modeling method. It captures both long-term and short-term dependencies in multimodal data with linear computational complexity. This improves computational efficiency and enables real-time performance.

The contributions of the research are presented in summary as follows:

- A temporal modeling method based on the RWKV architecture is proposed. It captures long-term and short-term dependencies in visual and inertial data efficiently. Its lightweight design reduces computational complexity.
- A novel IMU encoder, Res-Encoder, integrates convolutional layers with residual connections to enhance the extraction of inertial features. It effectively preserves historical information, enhancing the depth and stability of feature representations.
- A parallel IMU encoder structure is introduced to extract diverse features from inertial data, enhancing the expressiveness of inertial features and improving the fusion of multi-modal data.

Experimental results show that RWKV-VIO performs better than existing methods in terms of both localization accuracy and computational efficiency, fully demonstrating its robustness in complex environments and advantages in practical applications.

## 2. Related Work

In this section, we provide an overview of recent advancements in visual odometry (VO), visual–inertial odometry (VIO), and key supporting techniques for odometry, with a focus on deep learning-driven approaches. To systematically clarify the characteristics, technical paths, and limitations of representative methods in these areas, Table 1 first summarizes classic and influential works—including those discussed in subsequent VO and VIO sections—by category, sensor configuration, and core attributes. This structured comparison provides a foundational reference for analyzing gaps in existing research and highlighting the necessity of our proposed framework.

### 2.1. Visual Odometry

Existing VO research primarily includes traditional feature-based and deep learning-based approaches. Early feature-based methods relied on manually designed descriptors: ORB [19] was widely used for feature extraction and matching, enabling motion estimation via geometric triangulation, but failed in texture-less scenes and was sensitive to motion blur.

The shift to deep learning led to end-to-end VO models. DeepVO [30] used deep recurrent convolutional neural networks (DRCNNs) to learn motion directly from image sequences, eliminating manual feature engineering. Monodepth2 [34] introduced self-supervised learning for monocular depth estimation, supporting VO scale calibration. BeyondTracking [35] added a memory selection mechanism to refine long-sequence poses, while GFS-VO [37] used guided feature selection to enhance visual feature quality. However, these VO methods suffered from inherent limitations: monocular setups had scale ambiguity, leading to long-term drift, and pure visual data failed to compensate for feature loss in dynamic or low-light environments.

### 2.2. Visual-Inertial Odometry

To address VO’s gaps, VIO fuses visual data with IMU measurements, leveraging the camera’s spatial precision to suppress drift and the IMU’s high temporal resolution to fill visual feature gaps.

Early VIO methods were geometry-driven. ORB-SLAM3 [19] integrated IMU data into nonlinear optimization, supporting multisensor modalities for high accuracy in controlled environments. VINS-Mono [18] used a tightly coupled EKF to solve monocular scale ambiguity via IMU pre-integration. ROVIO [20] avoided feature extraction, using dense image alignment to fuse IMU data with pixel intensity for low-texture efficiency. However, these methods relied on manual parameter tuning and had high computational complexity, limiting real-time performance on resource-constrained platforms.

Deep learning-based VIO enabled end-to-end spatiotemporal learning. VINet [29]—the first end-to-end deep VIO model—used DeepVO’s [30] visual extractor and LSTM to process IMU data, projecting fused features into SE(3) space. Subsequent works optimized fusion: Chen et al. [32] proposed soft/hard fusion for corrupted inputs; ATVIO [36] used attention to balance visual–inertial streams and adaptive loss for pose regression. VIOLearner [31] added unsupervised training for trajectory correction, while DeepVIO [33] used 3D geometric constraints to suppress drift. Recent models like VIFT [38] adopted Transformers for long-range dependencies, and Fusion [32] refined selective fusion. Yet, limitations persisted: LSTM models had gradient vanishing in long sequences, Transformers had quadratic complexity, and many underutilized IMU data, leading to insufficient feature representation and dynamic environment inaccuracy.

### 2.3. Temporal Modeling

In addition to the advancements in VIO, significant progress has been made in temporal modeling. In recent years, innovative approaches to temporal modeling have significantly improved efficiency and performance through breakthroughs in architectural design. Transformer-based models [39,40,41] have demonstrated exceptional performance in handling long-sequence tasks due to their self-attention mechanism and parallel computing capabilities. However, their quadratic complexity limits their application in resource-constrained scenarios. In contrast, CNNs [42,43] have shown strong modeling capabilities in long-term sequence prediction tasks by effectively capturing local and global patterns in time series. Additionally, explorations of Multilayer Perceptrons (MLPs) in temporal modeling have also achieved remarkable progress. For instance, models like DLinear [44] achieve a performance comparable to more complex architectures with simple linear structures, prompting a reevaluation of the necessity of Transformer models. Recently, RNN-based architectures, such as RWKV [28], combine the global modeling capabilities of Transformers with the recursive nature of RNNs, exhibiting greater efficiency and competitiveness.

Traditional LSTM-based methods in VIO are limited by their sequential nature, resulting in slow inference speeds, which hinder real-time performance. Inspired by the efficiency of RWKV-TS [45], this work designs a lightweight temporal modeling framework that directly addresses these issues. By reducing the computational complexity of temporal dependency modeling, the proposed approach accelerates inference. It ensures a robust integration of IMU and visual data, making it suitable for dynamic environments and real-time applications.

## 3. Materials and Methods

**Figure 2:**
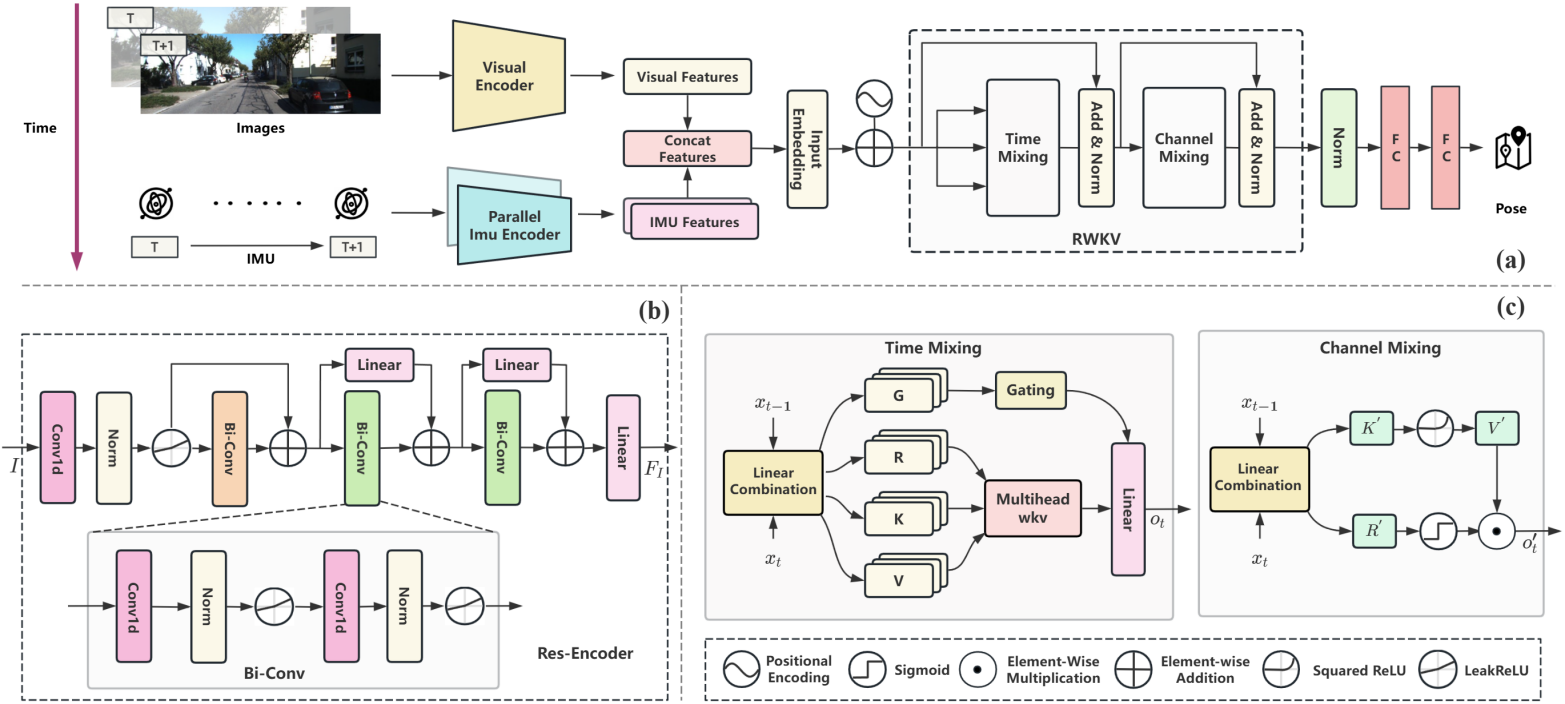
(**a**) RWKV-VIO Architecture. (**b**) The structure of the IMU encoder, which uses convolution and residual modules to extract temporal features from inertial data. (**c**) The RWKV module diagram with time mixing and channel mixing for modeling temporal and channel feature relationships.

We propose an in-depth description of the proposed deep learning-based VIO model. As illustrated in Figure 2a, the framework consists of a visual encoder, Res-Encoder, parallel encoder strategy, a positional encoding module, and a decoder built upon the RWKV network architecture. Each component is crafted to handle the specific challenges associated with visual–inertial fusion. This allows for effective feature extraction, reliable temporal modeling, and accurate pose estimation.

### 3.1. End-to-End Deep Learning-Based Visual–Inertial Odometry

The end-to-end Visual-Inertial Odometry (VIO) algorithm takes as input two consecutive monocular image frames along with a set of Inertial Measurement Unit (IMU) measurements recorded during the time interval between them. More comprehensively, for a VIO system, the inputs consist of the monocular video frames ${\left\{V_{i}\right\}}_{i=1}^{N}$, IMU measurements ${\left\{I_{i}\right\}}_{i=1}^{Nl}$ (captured with a sampling frequency *l* times higher than the video frame rate), and the initial camera pose $P_{1}$. Here, *N* denotes the total number of video frames, with i=1 representing the first frame and i=N the last frame. The goal of VIO is to estimate the camera poses ${\left\{P_{i}\right\}}_{i=2}^{N}$ for the entire path, where $V_{i}\in R^{3\times H\times W}$, $I_{i}\in R^{6}$, and $P_{i}\in \mathrm{SE}\left(3\right)$. The IMU provides six-dimensional measurements, which include three-dimensional linear accelerations (along the *x*-, *y*-, and *z*-axes of the IMU coordinate system) and three-dimensional angular velocities (around the *x*-, *y*-, and *z*-axes of the IMU coordinate system). These measurements reflect the instantaneous motion state of the device. One typical way to perform VIO is to estimate the six-DoF relative pose $T_{t\to t+1}$ that satisfies $P_{t}T_{t\to t+1}=P_{t+1}$ using two consecutive images $V_{t\to t+1}=\left\{V_{t},V_{t+1}\right\}$ and a set of IMU measurements $I_{t\to t+1}=\left\{I_{tl},\ldots ,I_{(t+1)l}\right\}$ for the time index t=1,2,…,N−1. The relative pose $T_{t\to t+1}$ can be further decomposed into a rotational component (represented by a rotational vector $\phi_{t}\in R^{3}$ containing Euler angles, which describe the orientation change) and a translational component (a translational vector $v_{t}\in R^{3}$, which represents the displacement in three-dimensional space). Our method learns a selection strategy that opportunistically skips the visual information $V_{t\to t+1}$ to reduce the computational complexity while maintaining the relative pose estimation accuracy.

During training, our method employs a sliding window approach, where image frames of length $seq_{-}len$ and their corresponding IMU measurements are inputted to the model at each step. This is attributed to the parallel computation capability of our pose regression network, which differs from LSTM’s approach of predicting the next pose based on the current pose. Our model predicts $seq_{-}len-1$ relative poses $\left\{P_{t\to t+seq_{-}len-1}\right\}$ in each step and then predicts the next set of $seq_{-}len-1$ relative poses in the following step, thus traversing the entire sequence.

### 3.2. Feature Encoder

#### 3.2.1. Visual Encoder

In this study, the classic FlowNet architecture [46] is adopted as the visual encoder to efficiently extract motion features, local textures, and robust global characteristics from image sequences. FlowNet employs a series of convolutional layers that progressively capture features at different levels of abstraction. Starting from basic elements such as edges and textures, the network advances to more complex motion patterns and spatial relationships. The architecture consists of multiple convolutional layers, each with increasing channel sizes, enabling the network to effectively model dynamic relationships within the scene. As shown in Figure 2a, by inputting consecutive image frames $V_{T}$ and $V_{T+1}$ into the optical flow estimation network to obtain visual features ($F_{V}$) with a dimensionality of 512:

(1) $$F_{V}=FlowNet\left(\left[V_{T};V_{T+1}\right]\right)$$

#### 3.2.2. IMU Encoder

The IMU encoder architecture, as depicted in Figure 2b, is based on a Res-Encoder (residual convolutional neural network) designed for effective inertial representation learning. The input to the encoder is a sequence $I\in R^{B\times S\times C\times L}$, where *B* denotes the batch size, *S* the temporal window length (e.g., number of video frames per sample), C=6 for the raw IMU channels (three-axis accelerometer and gyroscope), and *L* the number of IMU steps between two video frames.

The encoding process begins with an initial 1D convolution to extract low-level features from each IMU data channel:

(2) $$X_{0}=\phi_{\mathrm{init}}\left(I\right)=\mathrm{Drop}\left(\sigma \left(\mathrm{BN}\left(\mathrm{Conv}1d_{6\to 64}\left(I\right)\right)\right)\right),$$

where $\mathrm{Conv}1d_{6\to 64}$ denotes a 1D convolution with 64 filters, BN is batch normalization, σ is the LeakyReLU activation, and Drop is dropout for regularization. Here, $X_{0}$ is the output feature after initial 1D convolution of IMU data, and $\phi_{\mathrm{init}}$ is the initial feature extraction function integrating convolution, batch normalization, activation, and dropout.

Subsequently, the encoder employs a stack of three residual convolutional blocks, each denoted as Bi-Conv, to progressively extract higher-level temporal and channel features while suppressing noise. The *k*-th Bi-Conv block consists of two convolutional layers with possible channel expansion, and incorporates a residual (shortcut) connection to promote feature reuse and gradient flow:

(3) $$\begin{array}{cc} Z_{1}^{\left[k\right]} & =\sigma (\mathrm{BN}\left(\mathrm{Conv}1d_{C_{k}\to C_{k+1}}\left(X_{k-1}\right)\right)), \end{array}$$

(4) $$\begin{array}{cc} Z_{2}^{\left[k\right]} & =\sigma (\mathrm{BN}\left(\mathrm{Conv}1d_{C_{k+1}\to C_{k+1}}\left(Z_{1}^{\left[k\right]}\right)\right)), \end{array}$$

(5) $$\begin{array}{cc} Z_{2}^{\left[k\right]} & =\mathrm{Drop}(Z_{2}^{\left[k\right]}). \end{array}$$

where $Z_{1}^{\left[k\right]}$, $Z_{2}^{\left[k\right]}$ are intermediate feature tensors in the *k*-th residual block, σ denotes an activation function (e.g., LeakyReLU), BN is batch normalization, $\mathrm{Conv}1d_{C_{\mathrm{in}}\to C_{\mathrm{out}}}$ represents a 1D convolution with $C_{\mathrm{in}}$ input channels and $C_{\mathrm{out}}$ output channels, $X_{k-1}$ is the input feature tensor to the *k*-th block, and Drop is dropout for regularization. When the input and output channels differ ($C_{k}\neq C_{k+1}$), the residual path is projected via a 1×1 convolution:

(6) $$\begin{array}{cc} R^{\left[k\right]} & =\left\{\begin{array}{cc} X_{k-1}, & \mathrm{if}\;C_{k}=C_{k+1},\\ \mathrm{Conv}1d_{C_{k}\to C_{k+1}}\left(X_{k-1}\right), & \mathrm{if}\;C_{k}\neq C_{k+1}, \end{array}\right. \end{array}$$

(7) $$\begin{array}{cc} X_{k} & =Z_{2}^{\left[k\right]}+R^{\left[k\right]}. \end{array}$$

The three Bi-Conv blocks in sequence operate at channel dimensions $C_{1}=64,$ $C_{2}=128,\;C_{3}=256$, respectively. This hierarchical design enables the encoder to capture increasingly abstract inertial patterns across the temporal window and enhance robustness to sensor noise.

After the stacked residual blocks, the resulting feature tensor is flattened across channel and temporal dimensions, and then projected into a fixed-length latent vector to obtain the final IMU feature representation:

(8) $${\hat{F}}_{I}=\psi_{\mathrm{proj}}\left(X_{3}\right)=\mathrm{FC}\left(\mathrm{Flatten}(X_{3})\right),$$

where ${\hat{F}}_{I}$ is the final IMU feature representation, $\psi_{\mathrm{proj}}$ is the projection function, $X_{3}$ is the feature tensor from stacked residual blocks, “Flatten” reshapes $X_{3}$ into a vector, and FC projects it to dimension d=256.

To further enhance representation diversity and robustness, inspired by ensemble learning [47], a parallel architecture is employed. Specifically, two independently initialized Res-Encoders process the same IMU input in parallel:

(9) $$F_{I}=\left[{\hat{F}}_{I}^{\left(1\right)};{\hat{F}}_{I}^{\left(2\right)}\right]\in R^{B\times S\times 512},$$

where [·;·] denotes vector concatenation, and ${\hat{F}}_{I}^{\left(1\right)}$, ${\hat{F}}_{I}^{\left(2\right)}$ are the outputs of the parallel encoders. Such integration yields a 512-dimensional IMU feature per sample and temporal window, and ensures that inertial features are well-aligned with the extracted visual features for subsequent cross-modal fusion.

In summary, the IMU encoder adopts a deeply residual 1D convolutional backbone augmented with dimension-matching projections, dropout, and a parallel ensemble strategy, to extract expressive and robust inertial representations for deep VIO.

### 3.3. Decoder

To address the challenge of capturing both short- and long-term dependencies in time series modeling while meeting real-time requirements, this study designs a decoder module based on a single-layer RWKV network, as shown in Figure 2c. By incorporating positional encoding, time mixing, and channel mixing mechanisms, efficient time series feature modeling and decoding were achieved.

#### 3.3.1. Positional Encoding

To enable the model to perceive and leverage the temporal ordering of the multimodal sequence, we employ an explicit positional encoding mechanism that augments the fused feature representations with absolute position information. The inputs to the embedding module consist of visual features ($F_{V}$) and inertial features ($F_{I}$), concatenated along the feature dimension to form a unified multimodal representation:

(10) $$X_{\mathrm{concat}}=\mathrm{concat}\left(\left[F_{V},F_{I}\right]\right)$$

Next, to extract meaningful local temporal dependencies, we apply a one-dimensional convolutional layer to $X_{\mathrm{concat}}$, which is particularly effective for modeling short-range correlations and projecting the concatenated features into the embedding space:

(11) $$X_{\mathrm{feature}}=Conv1D\left(X_{\mathrm{concat}}\right)$$

Here, Conv1D(·) denotes the 1D convolution operation with learnable kernels, which can capture dynamic transitions within short windows of the sequential data [48], offering a rich representation for subsequent temporal modeling.

To encode absolute position information, we incorporate a fixed, non-learnable sinusoidal positional encoding scheme as originally proposed by Vaswani et al. [49]. For each time step pos (corresponding to the synchronized sensor data at that moment), and each dimension *i* of the embedding space of dimensionality *d*, the positional encoding is defined as

(12) $$\begin{array}{cc} PE_{\left(pos,\;2i\right)} & =\sin\left(\frac{pos}{10000^{2i/d}}\right) \end{array}$$

(13) $$\begin{array}{cc} PE_{\left(pos,\;2i+1\right)} & =\cos\left(\frac{pos}{10000^{2i/d}}\right) \end{array}$$

where pos is the temporal index of the sequence, and i∈{0,1,…,d/2−1}. This alternating use of sine and cosine functions at different frequencies allows the model to encode temporal ordering in a manner that facilitates generalization to sequence lengths and positions not seen during training, promoting both local and global awareness of sequence structure.

The final input embedding is produced by performing an element-wise addition between the feature-encoded representation and the positional encoding:

(14) $$X_{\mathrm{embed}}=X_{\mathrm{feature}}+PE$$

This operation ensures that each embedded feature vector not only contains multimodal semantic content but is also uniquely conditioned on its temporal position in the sequence. Such a design enables the network to utilize both local spatiotemporal features and explicit global order information, resulting in robust and contextually enriched descriptors for downstream tasks such as sequence modeling and temporal reasoning.

#### 3.3.2. Time-Mixing Block

The time-mixing module is used to model temporal dependencies. In RWKV, Token Shift is introduced as a simple method for temporal feature mixing. For the current time step $x_{t}$ and the previous time step $x_{t+1}$, mixed features are generated through a linear combination, as given by the following equations:

(15) $$\begin{array}{c} g_{t}=W_{g}\cdot \left(\mu_{g}⊙x_{t}+\left(1-\mu_{g}\right)⊙x_{t-1}\right), \end{array}$$

(16) $$\begin{array}{c} r_{t}=W_{r}\cdot \left(\mu_{r}⊙x_{t}+\left(1-\mu_{r}\right)⊙x_{t-1}\right), \end{array}$$

(17) $$\begin{array}{c} k_{t}=W_{k}\cdot \left(\mu_{k}⊙x_{t}+\left(1-\mu_{k}\right)⊙x_{t-1}\right), \end{array}$$

(18) $$\begin{array}{c} v_{t}=W_{v}\cdot \left(\mu_{v}⊙x_{t}+\left(1-\mu_{v}\right)⊙x_{t-1}\right), \end{array}$$

where $u_{g},u_{r},u_{k},u_{v}$ are trainable weight parameters used to dynamically adjust the influence ratio of the current timestep and the historical timestep; ⊙ denotes the Hadamard product (performing element-wise multiplication on vectors or matrices of the same dimension); and · denotes matrix multiplication (multiplies the weight matrix by the feature vector) when paired with weight matrices like $W_{g}$.

The above operations are applied over the entire sequence of length *T*, producing four temporal feature sequences:

(19) $$\begin{array}{cc} G & ={\left\{g_{t}\right\}}_{t=1}^{T},\;R={\left\{r_{t}\right\}}_{t=1}^{T},\\ K & ={\left\{k_{t}\right\}}_{t=1}^{T},\;V={\left\{v_{t}\right\}}_{t=1}^{T} \end{array}$$

These sequences form what we denote as the **GRKV** block, which is visualized in Figure 2c. Each path (G, R, K, V) represents the full set of corresponding vectors across all time steps. Specifically, *G* is used for gating, while *R*, *K*, and *V* are fed into the WKV attention mechanism to capture temporal dependencies. Thus, GRKV refers not to a single timestep, but to the collection of all $\left(g_{t},r_{t},k_{t},v_{t}\right)$ vectors over the sequence.

To further capture global temporal dependencies, the temporal mixing module employs a multi-head Weight-Key-Value (WKV) operation for global temporal modeling. The computation for a single-head WKV is defined as

(20) $$wkv_{t}=diag\left(u\right)\cdot k_{t}^{⊤}\cdot v_{t}+\sum_{i=1}^{t}diag{\left(w\right)}^{t-1-i}\cdot k_{t}^{⊤}\cdot v_{i}$$

where *u* represents the reward parameter for the current time step, *w* is the temporal decay vector (balancing the influence of the current time step and historical time steps), and diag(·) denotes the operator that converts a 1D vector into a diagonal matrix (vector elements serve as main diagonal entries, with all other entries set to 0). The temporal decay weights are updated iteratively and constrained to $w\in \left(0,1\right)$, ensuring that diag(w) is a contraction matrix:

(21) w=exp(−exp(w))

To enhance the expressiveness of the model, RWKV introduces a multi-head WKV mechanism, computed as

(22) $$\mathrm{Multiheadwk}v_{t}=\mathrm{Concat}\left(wkv_{t}^{1},\cdots ,wkv_{t}^{h}\right)$$

where *h* is the number of heads, and Concat(·) denotes the concatenation operator that merges multiple vectors along the feature dimension. In practical implementation, reshaping operations are used to optimize computational efficiency, completing the multi-head WKV calculation. The final output of the temporal mixing module is processed by the SiLU activation function and a normalization operation for each head, effectively performing GroupNorm [50] for *h* groups. Note that · in $r_{t}\cdot wkv_{t}$ denotes element-wise multiplication (multiplies corresponding elements of the feature vectors $r_{t}$ and $wkv_{t}$). The output is given by

(23) $$o_{t}=\left(SiLU\left(g_{t}\right)⊙LayerNorm\left(r_{t}\cdot wkv_{t}\right)\right)W_{o}$$

#### 3.3.3. Channel-Mixing Block

The channel-mixing module is designed to enhance feature interaction across channels, further processing temporal features through weighted combinations and non-linear transformations. The operations are as follows:

(24) $$\begin{array}{cc} \; & {k_{t}}^{\prime }={W_{g}}^{\prime }\cdot \left({\mu_{k}}^{\prime }⊙x_{t}+\left(1-{\mu_{k}}^{\prime }\right)⊙x_{t-1}\right), \end{array}$$

(25) $$\begin{array}{cc} \; & {r_{t}}^{\prime }={W_{r}}^{\prime }\cdot \left({\mu_{r}}^{\prime }⊙x_{t}+\left(1-{\mu_{r}}^{\prime }\right)⊙x_{t-1}\right), \end{array}$$

(26) $$\begin{array}{cc} \; & {v_{t}}^{\prime }=ReLU^{2}\left({k_{t}}^{\prime }\cdot {W_{v}}^{\prime }\right) \end{array}$$

(27) $$\begin{array}{cc} \; & {o_{t}}^{\prime }=Sigmoid\left({r_{t}}^{\prime }\right)⊙{v_{t}}^{\prime } \end{array}$$

where $ReLU^{2}\left(\cdot \right)$ denotes the squared ReLU operator (it first applies ReLU activation: ReLU(x)=max(0,x), then squares the activation result); ReLU is employed as a non-linear activation function to enhance the model’s expressive capability; and Sigmoid is used to regulate the influence of temporal features on the final output.

The above operations are applied across all timesteps in the sequence, generating three feature sequences:

(28) $$K^{\prime }={\left\{k_{t}^{\prime }\right\}}_{t=1}^{T},\;R^{\prime }={\left\{r_{t}^{\prime }\right\}}_{t=1}^{T},\;V^{\prime }={\left\{v_{t}^{\prime }\right\}}_{t=1}^{T}$$

These represent the channel-mixing counterparts to GRKV: $K^{\prime }$ encodes transformed features, $R^{\prime }$ acts as a gate, and $V^{\prime }$ contains nonlinearly activated values. They are combined at each time step to form the final output ${\left\{o_{t}^{\prime }\right\}}_{t=1}^{T}$. This process is performed in parallel across the entire sequence.

#### 3.3.4. Pose Regression Module

After processing by the RWKV module, the learned temporal and contextual features are forwarded to a dedicated pose regression network. This regression module consists of a simple yet effective two-layer fully connected neural network, whose structure is illustrated as follows:

$$\mathrm{nn}.\mathrm{Sequential}\left(\underset{\mathrm{feature}\;\mathrm{projection}}{\underset{︸}{\mathrm{nn}.\mathrm{Linear}(d_{in},\;128)}},\;\underset{\mathrm{nonlinearity}}{\underset{︸}{\mathrm{nn}.\mathrm{LeakyReLU}(0.1)}},\;\underset{\mathrm{pose}\;\mathrm{output}}{\underset{︸}{\mathrm{nn}.\mathrm{Linear}(128,\;6)}}\right)$$

Given the high-dimensional embedded features $X_{embed}\in R^{d_{in}}$ from the RWKV module, the first linear layer projects the input to a 128-dimensional latent space. This serves to condense and distill the most salient information necessary for regression, enabling more effective learning. A LeakyReLU activation function with a negative slope of 0.1 is then applied to introduce nonlinearity and mitigate issues such as the dying ReLU problem, thus increasing the model’s expressive capacity while allowing a small gradient when the unit is not active.

Following this, a final linear transformation maps the 128-dimensional latent representation to a six-dimensional output vector, which corresponds to the target pose parameters: translation (x,y,z) and rotation $\left(r_{x},r_{y},r_{z}\right)$. These six values represent the location and orientation of the object at the predicted future time step.

The overall regression operation can be formally written as

(29) $$P_{t\to t+seq_{-}len-1}=R\left(RWKV(X_{embed})\right)$$

where R(·) denotes the two-layer regression module described above, and $P_{t\to t+seq_{-}len-1}$ encodes the predicted six-DoF pose (three translations and three rotations) from time step *t* to $t+seq_{-}len-1$, providing both the position and attitude information required for pose estimation tasks.

To summarize, although the regression network adopts a straightforward, fully connected design, its careful dimensionality reduction and the inclusion of nonlinear activation allow it to precisely transform the temporally enriched features from the RWKV module into accurate pose estimates.

### 3.4. Loss Function

During training, we use the Mean Squared Error (MSE) loss function, while also introducing a weight factor to balance the rotation and translation errors. The loss is defined as

(30) $$L_{pose}=\frac{1}{T-1}\sum_{t=1}^{T-1}\left({∥{\hat{v}}_{t}-v_{t}∥}_{2}^{2}+\alpha {∥{\hat{\phi }}_{t}-\phi_{t}∥}_{2}^{2}\right)$$

where *T* is the sequence length, ${\hat{v}}_{t}$ and ${\hat{\phi }}_{t}$ represent the predicted translation and rotation parameters, while $v_{t}$ and $\phi_{t}$ denote the ground truth translation and rotation parameters. α is a weight factor that is used to balance the scale of translation and rotation errors.

## 4. Results

We executed extensive experiments to measure the performance and merits of the RWKV module. These evaluations include main results, ablation studies, and detailed analyses of computational efficiency and parameter count. We further analyzed the experimental findings to provide a deeper insight into the effectiveness of the proposed approach.

### 4.1. Experiment Setup

#### 4.1.1. Dataset

**Figure 3:**
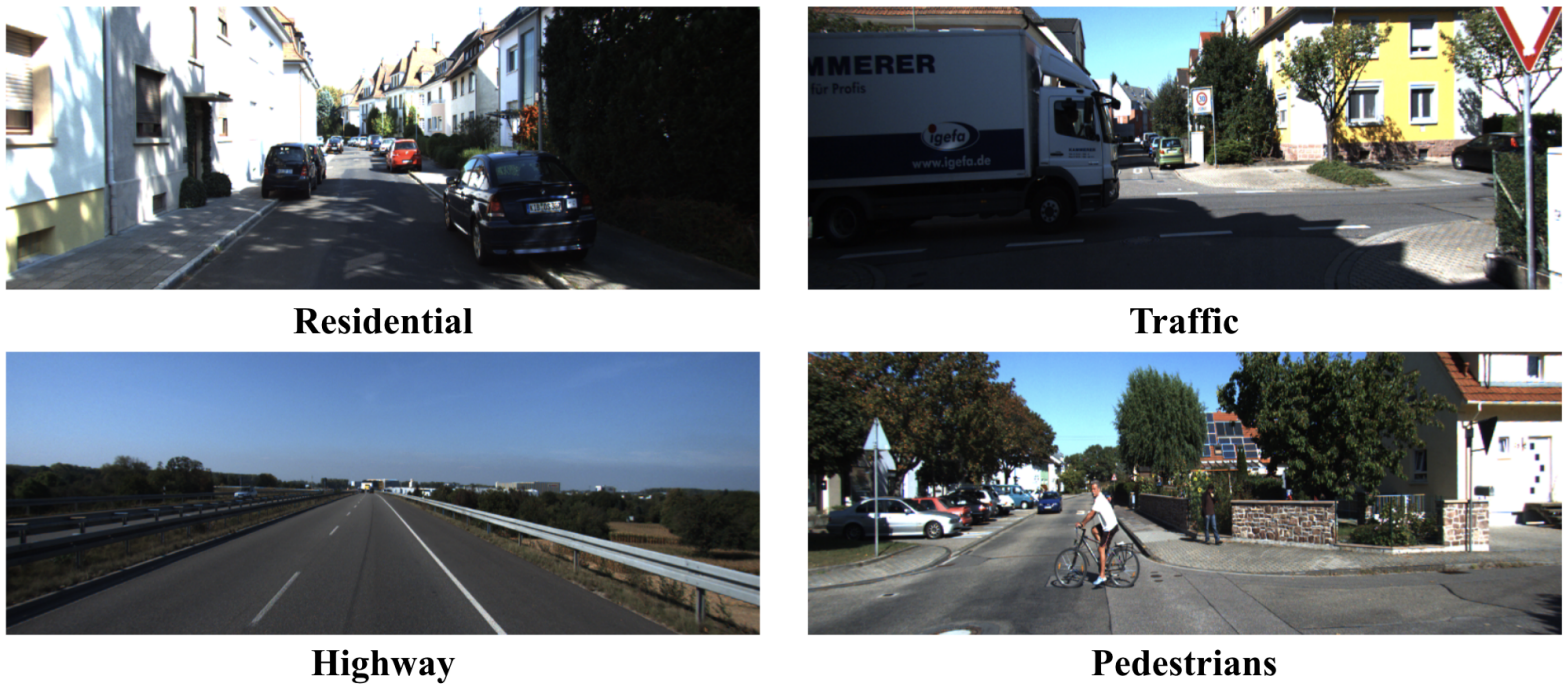
Examples of the KITTI Odometry dataset displaying scenes of residential areas, highways, hard traffic roads, and scenes with pedestrians.

This study uses the KITTI Odometry dataset [51] as the experimental benchmark, which is a widely recognized evaluation standard in the visual/visual–inertial odometry (VO/VIO) domain. The dataset was carefully collected by a real vehicle. The vehicle was equipped with a set of advanced sensors, including cameras, a 3D lidar, a GPS navigation system, and an IMU sensor. As shown in Figure 3, the vehicle was driven through various real-world scenarios, such as densely populated urban areas, serene rural areas, high-speed highways, quiet residential neighborhoods, areas with different weather and traffic conditions, and campus environments. The dataset contains 22 sequences. Among them, sequences 00 to 10 supply ground-truth pose data for the training phase, whereas sequences 11 to 22 lack ground-truth pose data and are exclusively employed for evaluation. We selected sequences 00, 01, 02, 04, 06, 08, and 09 for training and sequences 05, 07, and 10 for testing. Since sequence 03 lacks IMU data, it was excluded from the experiments.

The images and ground-truth poses in the dataset are sampled at a frequency of 10 Hz, while the IMU data are sampled at 100 Hz. To address temporal misalignment between the IMU data, images, and ground-truth poses, we interpolate the raw IMU data to achieve temporal synchronization with the images and ground-truth poses. For data alignment, we handle the frequency difference between visual and inertial sensors: given that images are captured at 10Hz and IMU data is sampled at 100 Hz, each image frame is mapped to 10 consecutive IMU measurements to synchronize these modalities. For an image sequence of length $seq_{-}len$, the corresponding IMU data segment is extracted from the i×10-th to the (i+10)×10+1-th data point(where *i* denotes the starting index of the sequence), ensuring equidistant temporal alignment across the sequence. This indexing strategy maintains consistent time-series correspondence between images, their associated poses, and IMU data, as commonly adopted in visual–inertial odometry research on the KITTI dataset.

In the experiments, only monocular images from the left camera of the KITTI dataset are utilized as input data. The $t_{rel}$ and $r_{rel}$ are the main metrics used in the experiments, representing the average translational error and average rotational error of all subsequences with lengths ranging from 100 to 800 m.

#### 4.1.2. Implementation Details

During the training process, all images are resized to 512×256, and seq_len is set to 11. The visual encoder utilizes the FlowNet-S network pre-trained on the FlyingChairs [52] dataset (with the final layer removed) for optical flow feature extraction. The subsequent processing details and the inertial encoder were described earlier. Training employs a 60-epoch two-stage strategy with the Adam optimizer (weight decay $5\times 10^{-6}$, betas (0.9, 0.999), eps $1\times 10^{-8}$): the first 20 epochs use a warm-up learning rate of $5\times 10^{-4}$ to stabilize initial training, followed by 40 fine-tuning epochs with a reduced learning rate of $1\times 10^{-4}$ for parameter refinement. A fixed training schedule is adopted without early stopping, with checkpoints saved every 10 epochs and the final model weights from the 60th epoch used for evaluation. All experiments are executed on an NVIDIA RTX 4090 GPU (24G) with a batch size of 16, balancing training efficiency and model convergence.

### 4.2. Main Results

Table 2 summarizes the pose estimation results of various methods on the KITTI dataset. The proposed RWKV-VIO model demonstrates excellent overall performance, achieving an average translational error of 2.29% and an average rotational error of 1.26° over trajectory length. Specifically, RWKV-VIO ranks second in $t_{rel}\left(\%\right)$ for Sequence 05, and consistently remains among the top three in both Sequences 07 and 10 in terms of both translational and rotational errors. On average, RWKV-VIO attains the third-best results in both metrics, highlighting the model’s robustness and accuracy on different sequences.

Compared to other methods, RWKV-VIO ranks third on average in terms of accuracy. Although the overall average translational error and rotational error of RWKV-VIO are slightly higher than those of DeepVIO [33] and ORB-SLAM2 [19], it outperforms both methods in their weaker areas. DeepVIO achieves a lower rotational error, but suffers from a higher translational error, while ORB-SLAM2 performs well in rotation, but has a significantly higher translational error. In contrast, RWKV-VIO balances these two metrics effectively, achieving a lower translational error than ORB-SLAM2 and a better rotational error than DeepVIO. This balanced performance makes RWKV-VIO stand out in tasks where both translational and rotational errors are critical. Moreover, RWKV-VIO has a faster inference speed than almost all other models, making it a more practical choice for real-world applications.

### 4.3. Ablation Study

IMU measurements are affected by various sources of error, such as bias drift, temperature sensitivity, random sensor noise, and random walk. These errors can be significantly amplified during the integration phase of conventional inertial navigation algorithms, leading to trajectory drift and the accumulation of positioning errors. This degradation in accuracy becomes more pronounced over time and distance. In environments with poor visual textures, weak visual features, or even complete absence of visual cues, visual-based localization methods are severely limited. Under these challenging conditions, the reliance on IMU data becomes increasingly important, and the accuracy of IMU measurements has a direct impact on the reliability of the entire localization system.

**Figure 4:**
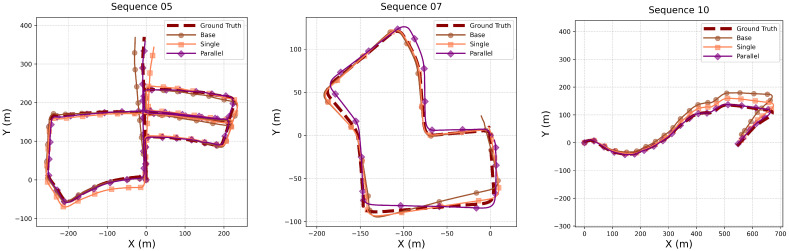
The results of the RWKV-VIO model’s positioning on KITTI Sequences 05 (**left**), 07 (**middle**), and 10 (**right**) with different encoder strategies.

Ablation experiments were conducted to evaluate the effectiveness of the proposed inertial encoder in reducing the trajectory drift and cumulative errors. These experiments were performed in consistent settings, with modifications applied only to the inertial encoder. Specifically, three configurations were tested: a baseline encoder consisting of three convolutional layers, a single Res-Encoder, and the proposed parallel encoder strategy. The baseline encoder processes six-channel IMU data through three sequential 1D convolutional layers with channel dimensions increasing from 6 to 64, 128, and 256 respectively, each followed by BatchNorm, LeakyReLU (negative slope 0.1), and Dropout, before projecting features to the target dimension via a fully connected layer. As illustrated in Figure 4, the parallel encoder strategy significantly improved performance. The trajectory in Sequence 10 exhibited marked convergence, while the trajectory in Sequence 05, particularly near the end, aligned more closely with the ground truth (GT) compared to both the baseline encoder and the single Res-Encoder.

**Figure 5:**
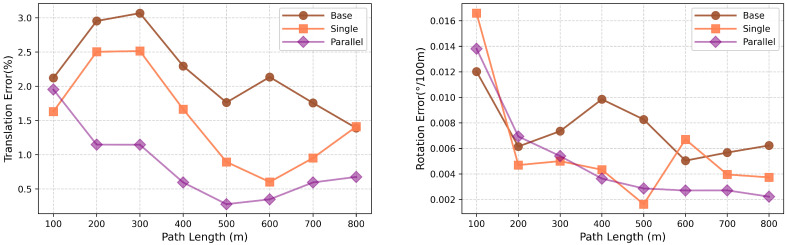
Average errors of RWKV-VIO on KITTI Sequence 05: Translation error ((**left**), %) and rotation error ((**right**), °/100 m) under different encoder strategies.

To validate the drift suppression efficacy of the proposed strategy, we evaluated average translation and rotation errors across distances on KITTI Sequence 05. As shown in Figure 5, the baseline encoder lacks residual connections and loses critical historical IMU features, resulting in notable error accumulation over distance and abrupt initial translation errors. The single Res-Encoder preserves partial shallow features via residuals yet shows distinct initial translation fluctuations and cumulative error trends. In contrast, the parallel strategy uses dual independent Res-Encoders for complementary IMU feature extraction, effectively reducing error accumulation, enhancing curve stability, and suppressing initial translation abruptness, which demonstrates that residual connections and parallel structures collectively enable robust suppression of IMU error accumulation.

As summarized in Table 3, the parallel strategy reduced the translation error by 31.8 % and the rotation error by 20.2 %, demonstrating substantial accuracy improvements. The use of multiple independently initialized IMU encoders enables the model to capture complementary features, improving robustness and reducing the risk of overfitting, similarly to an ensemble learning approach. Additionally, the parallel architecture aligns on the IMU and visual features, enabling a more effective fusion of temporal and spatial information. This design significantly mitigates short- and long-term trajectory drift, resulting in improved overall pose estimation accuracy.

### 4.4. Efficiency Analysis

This section begins by presenting a comparison of the time and space complexity of various models. As shown in Table 4, RWKV is a linear RNN model with time and space complexity of O(L), where *L* is the sequence length. In contrast, for models based on LSTM, the model presented by Yang et al. [22] can be considered a typical example. And the time complexity of LSTM is O(L). However, its processing is sequential, with the output of one timestep relying on the previous one. Therefore, when inferring a sequence of length *L*, the number of inference steps for LSTM is *L*, as reflected in Table 4. For models based on the Transformer, VIFT is a representative example. Transformer models, due to their self-attention mechanism, have a time complexity of $O(L^{2})$, which escalates rapidly as the sequence length increases, resulting in high computational costs.

**Figure 6:**
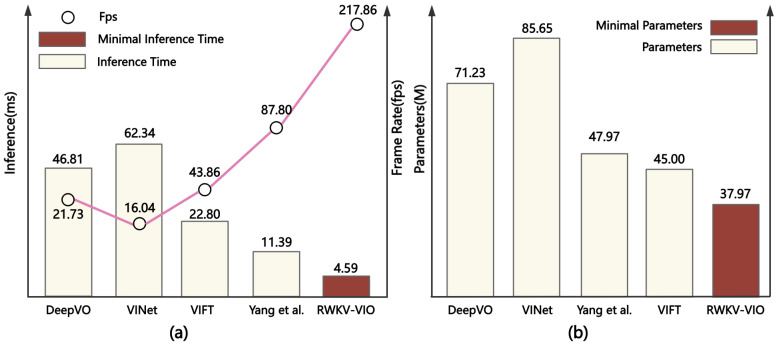
(**a**) A comparison of inference time and frame rate among different models. (**b**) The frame rate and parameter quantity of these models.

As shown in Figure 6a, we compared the inference time and frame rates of various models. RWKV-VIO achieves the best performance, with an inference time of only 4.59 ms and a frame rate of 217.86 fps, significantly outperforming DeepVO (46.81 ms, 21.73 fps), VINet (16.04 ms, 62.34 fps), VIFT (22.80 ms, 43.86 fps) and Yang et al.’s method (11.39 ms, 87.80 fps). In Figure 6b, RWKV-VIO has a parameter size of only 37.97 M, much smaller than DeepVO (71.23 M), VIFT (45 M), VINet (47.97 M) and the Yang et al. [22] method (85.65 M). This shows that RWKV-VIO excels both in inference efficiency and in terms of the lightweight design of the model.

The remarkable advantage of RWKV-VIO in inference time stems from the unique architecture of the RWKV algorithm. Traditional models like DeepVO and VINet rely on LSTM networks for temporal sequence processing. LSTM requires recursive computations, with each step depending on the previous state, leading to a serialized inference process. Transformer-based models such as VIFT have high accuracy. However, due to their self-attention mechanism that involves computing correlations between every position in the sequence and all others, the computational complexity soars as the sequence length increases. Consequently, their inference efficiency is affected. For example, VIFT’s inference speed is 22.8 ms, which is not particularly fast.

In contrast, RWKV avoids recursion by using a key-value mechanism to capture temporal features efficiently, enabling RWKV-VIO to achieve an inference speed of 4.59 ms, making it one of the fastest models currently available.

In terms of parameter size, RWKV-VIO also performs exceptionally well, with its parameter count being nearly half that of the Selective method. This is because RWKV reduces redundant parameters by optimizing its architecture, without relying on larger hidden dimensions or deeper networks as LSTM-based models do to compensate for their limitations in long-sequence dependency modeling. Additionally, RWKV aligns temporal and spatial features directly during fusion, reducing model complexity while maintaining strong feature representation capabilities.

## 5. Conclusions

In conclusion, the proposed RWKV-VIO framework introduces a novel approach to visual–inertial odometry, combining the strengths of the RWKV architecture with efficient temporal modeling. Using a lightweight structure with linear computational complexity, RWKV-VIO effectively captures both short- and long-term dependencies, overcoming the limitations of traditional recurrent models such as LSTMs and Transformers. The introduction of the parallel IMU encoder further enhances the feature extraction process, significantly improving performance in complex environments. Experimental findings demonstrate that RWKV-VIO performs better than existing methods when it comes to localization accuracy, computational efficiency, and real-time performance. The framework improves both translational and rotational accuracy in a balanced manner, making it a reliable solution for real-world applications in autonomous navigation and robotics.

## Figures and Tables

**Table 1:** Classification and comparison of representative VO/VIO methods.

Method Category	Model	Year	Sensor	Keywords
**Geometry-based**	ORB-SLAM2 [19]	2021	Mono/RGB-D/Stereo	ORB feature tracking; nonlinear optimization
	VINS-Mono [18]	2018	Mono + IMU	Tight mono-IMU coupling; EKF-based estimation
	ROVIO [20]	2015	Mono + IMU	Direct EKF; robust inertial fusion
**Deep Learning-based**	VINet [29]	2017	Mono + IMU	End-to-end seq2seq; LSTM fusion
	DeepVO [30]	2017	Mono	DRCNNs; end-to-end VO
	VIOLearner [31]	2018	Mono + IMU	Unsupervised learning; online trajectory correction
	Chen et al. [32]	2019	Mono + IMU	Selective sensor fusion; neural network
	DeepVIO [33]	2019	Mono + IMU	Self-supervised learning
	Monodepth2 [34]	2019	Mono	Self-supervised depth
	BeyondTracking [35]	2019	Mono	Memory selection; pose refinement
	Zou et al. [27]	2020	Mono	Self-supervised long-term; CNN-RNN hybrid
**Deep Learning-based**	ATVIO [36]	2021	Mono + IMU	Attention fusion; adaptive loss
	GFS-VO [37]	2018	Mono	Guided feature selection
	Tu et al. (EMA-VIO) [23]	2022	Mono + IMU	External memory attention
	Yang et al. [22]	2022	Mono + IMU	Adaptive visual modality; LSTM modeling
	VIFT [38]	2024	Mono + IMU	Causal Transformer
	Fusion [32]	2025	Mono + IMU	Selective fusion

**Table 2:** Comparison of VO/VIO methods categorized into geometry-based and learning-based approaches.

Method	Model	Type	Seq. 05		Seq. 07		Seq. 10		avg $t_{\mathrm{rel}}\left(\%\right)$	avg $r_{\mathrm{rel}}{(}^{\circ })$
			$t_{\mathrm{rel}}\left(\%\right)$	$r_{\mathrm{rel}}{(}^{\circ })$	$t_{\mathrm{rel}}\left(\%\right)$	$r_{\mathrm{rel}}{(}^{\circ })$	$t_{\mathrm{rel}}\left(\%\right)$	$r_{\mathrm{rel}}{(}^{\circ })$		
**Geometry**	ORB-SLAM2 [19]	VO	9.12	**0.2**	10.34	**0.3**	4.04	**0.3**	7.8	**0.27**
	VINS-Mono [18]	VIO	11.6	1.26	10.0	1.72	16.5	2.34	12.7	1.77
	ROVIO [20]	VIO	**3.21**	1.22	**2.97**	1.38	**3.20**	1.33	**3.13**	1.31
**Learning**	Monodepth2 [34]	VO	4.66	1.7	4.58	2.6	7.73	3.4	5.65	2.56
	Zou et al. [27]	VO	2.63	**0.5**	6.43	2.1	5.81	1.8	4.95	1.46
	VIOLearner [31]	VIO	3.00	1.40	3.60	2.06	2.04	1.37	2.88	1.61
	DeepVIO [33]	VO	2.86	2.32	*2.71*	1.66	**0.85**	*1.03*	2.14	1.67
	GFS-VO [37]	VO	3.27	1.6	3.37	2.2	6.32	2.3	4.32	2.03
	DeepVO [30]	VO	2.62	3.61	3.91	4.60	8.11	8.83	4.88	5.58
	BeyondTracking [35]	VO	*2.59*	1.2	3.07	1.8	3.94	1.7	3.2	1.56
	ATVIO [36]	VIO	4.93	2.4	3.78	2.59	5.71	2.96	4.8	2.65
	Fusion [32]	VIO	4.44	1.69	2.95	1.32	3.41	1.41	3.02	1.42
	Yang et al. [22]	VIO	2.61	1.06	1.83	*1.35*	3.11	1.12	2.55	1.17
	VIFT [38]	VIO	**2.02**	0.53	**1.75**	**0.47**	2.57	**0.54**	**2.01**	**0.71**
	(Ours) RWKV-VIO	VIO	2.03	*1.0*	2.73	1.79	*2.1*	0.99	*2.29*	*1.26*

**bold** indicates the highest score, underlined indicates the second-highest score, and *italicized* indicates the third-highest score for each block.

**Table 3:** Ablation study results on imu encoder.

Method	Seq. 05		Seq. 07		Seq. 10		avg $t_{\mathrm{rel}}\left(\%\right)$	avg $r_{\mathrm{rel}}{(}^{\circ })$
	$t_{\mathrm{rel}}\left(\%\right)$	$r_{\mathrm{rel}}{(}^{\circ })$	$t_{\mathrm{rel}}\left(\%\right)$	$r_{\mathrm{rel}}{(}^{\circ })$	$t_{\mathrm{rel}}\left(\%\right)$	$r_{\mathrm{rel}}{(}^{\circ })$		
**base**	3.38	1.25	**2.65**	**1.70**	4.04	1.85	3.36	1.58
**+new imu encoder**	2.76	1.13	2.99	1.81	3.13	1.56	2.96	1.50
**+parallel imu encoder**	**2.03**	**1.00**	2.73	1.79	**2.13**	**0.99**	**2.29**	**1.26**

**bold** indicates the highest score, underlined indicates the second-highest score.

**Table 4:** Time complexity and space complexity comparison on LSTM, Transformer and RWKV.

Method	Model	Time	Space	Inference Step
LSTM	Yang et al. [22]	O(L)	O(L)	*L*
Transformer	VIFT [38]	$O(L^{2})$	$O(L^{2})$	*L*
RWKV	RWKV-VIO	O(L)	O(L)	1

## Data Availability

The data used in this study is derived from the KITTI dataset, which is publicly available at its official website: http://www.cvlibs.net/datasets/kitti/ (accessed on 9 September 2025).
